# PERCEIVED EXPERIENCES OF MINORITIZED FAMILY DEMENTIA CAREGIVERS IN MENTALIZING IMAGERY THERAPY

**DOI:** 10.1093/geroni/igae098.3380

**Published:** 2024-12-31

**Authors:** Miranda Zea, Paulina Gutierrez-Ramirez, Liliana Ramirez Gomez, Kimberly Dueck, Maria Melero-Dominguez, Felipe Jain

**Affiliations:** Massachusetts General Hospital, Boston, Massachusetts, United States; Massachusetts General Hospital, Boston, Massachusetts, United States; Massachusetts General Hospital, Boston, Massachusetts, United States; Massachusetts General Hospital, Boston, Massachusetts, United States; Massachusetts General Hospital, Boston, Massachusetts, United States; Depression Clinical and Research Program / Massachusetts General Hospital, Boston, Massachusetts, United States

## Abstract

Family dementia caregivers often experience stress and caregiver burden as a result of their responsibilities. Specifically, family dementia caregivers from racial and same sex minoritized groups face challenges including a lack of access to culturally concordant resources. Mentalizing Imagery Therapy (MIT) is a new mindfulness and guided imagery therapy that is designed to enhance self and other understanding, and alleviate psychological symptoms. We aimed to describe the perceived impact of MIT on three minoritized caregivers: a Black female, an Asian male, and a White gay female partnered to a Black woman. Across virtual and in-person trials of MIT (n=99), the proportion of racial and ethnic minority individuals was ~70%, and at least 4% sexual minorities. MIT comprised four, weekly, two-hour sessions, incorporating mindfulness and imagery exercises. Semi-structured exit interviews were conducted for perceived experiences by two independent raters, and consensus achieved. We purposefully selected three participants who could clearly express their thoughts and who described cultural factors for in-depth examination. Individuals from minoritized groups participating in MIT reported experiencing positive changes in well-being and an increased capacity for mentalization in relation to themselves and others (particularly the person living with dementia). They also reported gaining insights into unique cultural factors that impacted caregiving. These results suggest potential promise of MIT for aiding the distinct challenges confronted by minoritized family dementia caregivers. Participants demonstrated heightened well-being, and an increased capacity for mentalization. Future research should validate these findings with minority populations in larger samples.

